# Pharmacogenomic findings from clinical whole exome sequencing of diagnostic odyssey patients

**DOI:** 10.1002/mgg3.283

**Published:** 2017-03-19

**Authors:** Margot A. Cousin, Eric T. Matey, Patrick R. Blackburn, Nicole J. Boczek, Tammy M. McAllister, Teresa M. Kruisselbrink, Dusica Babovic‐Vuksanovic, Konstantinos N. Lazaridis, Eric W. Klee

**Affiliations:** ^1^ Center for Individualized MedicineMayo ClinicRochesterMinnesota; ^2^ Department of Health Sciences ResearchMayo ClinicRochesterMinnesota; ^3^ Center for Individualized MedicineMayo ClinicJacksonvilleFlorida; ^4^ Department of Health Sciences ResearchMayo ClinicJacksonvilleFlorida; ^5^ Department of Clinical GenomicsMayo ClinicRochesterMinnesota; ^6^ Department of GastroenterologyMayo ClinicRochesterMinnesota

**Keywords:** Exome sequencing, pediatric, pharmacogenomics, precision medicine, secondary findings

## Abstract

**Background:**

We characterized the pharmacogenomics (PGx) results received by diagnostic odyssey patients as secondary findings during clinical whole exome sequencing (WES) testing as a part of their care in Mayo Clinic's Individualized Medicine Clinic to determine the potential benefits and limitations to this cohort.

**Methods:**

WES results on 94 patients included a subset of PGx variants in *CYP2C19*,*CYP2C9*, and *VKORC1* if identified in the patient. Demographic, phenotypic, and medication usage information was abstracted from patient medical data. A pharmacist interpreted the PGx results in the context of the patients’ current medication use and made therapeutic recommendations.

**Results:**

The majority was young with a median age of 10 years old, had neurological involvement in the disease presentation (71%), and was currently taking medications (90%). Of the 94 PGx‐evaluated patients, 91% had at least one variant allele reported and 20% had potential immediate implications on current medication use.

**Conclusion:**

Due to the disease complexity and medication needs of diagnostic odyssey patients, there may be immediate benefit obtained from early life PGx testing for many and most will likely find benefit in the future. These results require conscientious interpretation and management to be actionable for all prescribing physicians throughout the lifetime of the patient.

## Introduction

Recent advances in genetics have provided benefit to individuals with inherited disease through the increasing availability of next generation sequencing (NGS) assays. Clinical whole exome sequencing (WES) tests result in a diagnosis for 25–30% of individuals with rare undiagnosed disease (Yang et al. 2013, 2014; Lee et al. 2014; Zhu et al. 2015; Lazaridis et al. 2016; Retterer et al. 2016). These patients on a diagnostic odyssey often have years from the onset of symptoms until they achieve a genetic diagnosis. WES is increasingly being used to evaluate diagnostic odyssey patients to identify the genetic cause of disease when traditional diagnostic testing has failed to resolve the etiology of disease or the symptoms of the patient do not suggest a likely diagnosis. WES interrogates sequence variation across protein‐coding regions of the genome, providing an expansive testing platform for diagnosing congenital conditions. WES also allows results secondary to the primary test indication to be reported, including genetic variation known to affect medication efficacy and toxicity. The impact and utility of these secondary results has been understudied in this unique population of diagnostic odyssey patients.

Pharmacogenomics (PGx), the study of genetic contribution to variability in drug response (Weinshilboum 2003; Weinshilboum and Wang 2004; Wang et al. 2011), has benefited from advances in testing platforms as well as the knowledge of genetic variations contributing to specific drug responses. PGx is increasingly utilized clinically to impact treatment decisions in a growing number of patients, with the majority of patients tested having at least one PGx allele that could affect the medication(s) efficacy or toxicity (Ji et al. 2016). The Food and Drug Administration has issued black‐box warnings on several medications with gene‐drug interactions, and precautions about others (www.fda.gov/drugs/scienceresearch/researchareas/pharmacogenetics/ucm083378.htm). Currently, >20 genes impact approximately 80 medications with clinical actionability (Relling and Evans 2015). PGx testing is often ordered for adults taking or being prescribed medications impacted by a known PGx gene. Knowledge of an individual's PGx genotypes could decrease the risk of major adverse drug reactions and improve therapeutic response (Relling and Evans 2015).

Secondary PGx results from diagnostic WES testing are often findings of convenience. Genes easily interrogated by NGS technology (e.g., *CYP2C9*, MIM:601130) with the majority of informative variants in the coding region, are easy to identify from WES data. However, for genes such as *CYP2D6* (MIM124030), standard WES does not perform well and it is difficult to achieve highly accurate and informative results (Kramer et al. 2009; Black et al. 2012; Ji et al. 2016). Limiting PGx testing due to technical challenges may lead to an incomplete profile, minimizing the therapeutic benefit achieved by comprehensive testing. This is particularly relevant to medications metabolized by more than one pharmacogene.

The context in which PGx findings are reported in clinical WES testing is arguably dissimilar to a standalone PGx test. The PGx findings in a WES test are secondary to the variants identified in disease‐causal genes that may explain the patient's symptomatology and, therefore, may be overlooked. Reported variants related to the primary genetic condition are already challenging to interpret and explain to the patient, making it even more onerous to put due focus on secondary results. Also, the physician ordering the WES test may not be the physician prescribing the patient's medications, adding another layer of complexity to the management and effective use of the PGx findings. WES test reports are often received as scanned static documents, and integrating this data into a record system capable of alerting prescribing physicians of pertinent PGx results is a significant need. PGx results for diagnostic odyssey patients, thus, have the potential to be overlooked with regard to current or future medication prescribing.

To assess the utility of the PGx secondary findings in clinical WES testing, we reviewed a cohort of individuals evaluated in Mayo Clinic's Individualized Medicine Clinic for undiagnosed disease and tested via clinical WES for the purpose of achieving a genetic diagnosis. Here, we report the PGx findings of this cohort, the immediate implications of these results on medication usage, and the unique characteristics and nuances associated with the appropriate management of these data. To the best of our knowledge, this is the first study evaluating the benefit of secondary PGx findings reported by a clinical WES test for patients seeking a genetic diagnosis.

## Materials and Methods

### Ethical compliance

The Mayo Clinic Institutional Review Board granted a waiver of consent for this study. To this end, it was the responsibility of the corresponding author of the study and/or his designee to check a patient's Minnesota research authorization status before reviewing any medical records generated from care received in the state of Minnesota for all patients included in this study. No patient included in this study declined Minnesota research authorization.

### Patients

All patients included in this study were referred to Mayo Clinic's Individualized Medicine Clinic for a suspected genetic disorder, were evaluated by a medical geneticist, and counseled by a genetic counselor prior to pursuing WES for the purpose of elucidating the genetic etiology of disease. Each patient's current medication usage and demographics were abstracted from chart review. The patient’s genetic disease phenotypes were abstracted from the clinical WES report, as reported by the ordering clinical geneticist.

### WES and PGx testing

Whole exome sequencing was conducted clinically through Baylor Genetics, Houston, Texas. The genes and variant alleles reported in this clinical WES test include *CYP2C19* (NG_008384.2; NC_000010.10; NM_000769.2; Build GRCh37.p13) alleles: *2, *3, *4, *5, *8, *10, and *17,* CYP2C9* (NG_008385.1; NC_000010.10; NM_000771.3; Build GRCh37.p13) alleles: *2, *3, *5, and *6, and the *VKORC1* (NG_011564.1; NC_000016.9; NM_024006; Build GRCh37.p13) allele: c.‐1639G>A and Baylor reports >20× coverage for 100% of the *CYP2C9*,* CYP2C19*, and *VKORC1* genes.

Reported Baylor Genetics Methodology for Whole Exome Sequencing:

1. “Whole exome sequencing (WES): for the paired‐end precapture library procedure, genome DNA is fragmented by sonicating genomic DNA and ligating to the Illumina multiplexing PE adapters. The adapter‐ligated DNA is then PCR amplified using primers with sequencing barcodes (indexes). For target enrichment/exome capture procedure, the precapture library is enriched by hybridizing to biotin‐labeled VCRome 2.1 in‐solution exome probes (Bainbridge et al. 2011) at 47°C for 64–72 h. Additional probes for over 3600 Mendelian disease genes were also included in the capture in order to improve the exome coverage. For massively parallel sequencing, the postcapture library DNA is subjected to sequence analysis on Illumina HiSeq platform for 100 bp paired‐end reads. The following quality control metrics of the sequencing data are generally achieved: >70% of reads aligned to target, >95% target base covered at >20X, >85% target base covered at >40X, mean coverage of target bases >100X. SNP concordance to genotype array: >99%. This test may not provide detection of certain genes or portions of certain genes due to local sequence characteristics or the presence of closely related pseudogenes. Gross deletions or duplications, changes from repetitive sequences may not be accurately identified by this methodology.
2. As a quality control measure, the individual's DNA is also analyzed by a SNP‐array (Illumina HumanExome‐12v1 array). The SNP data are compared with the WES data to ensure correct sample identification and to assess sequencing quality.
3. Data analysis and interpretation by Mercury: The output data from Illumina HiSeq are converted from bcl file to FastQ file by Illumina CASAVA 1.8 software (Illumina, San Diego, California, USA), and mapped by BWA program to the reference haploid human genome sequence (Genome Reference Consortium human genome build 37, human genome 19). The variant calls are performed using Atlas‐SNP and Atlas‐indel developed in‐house by BCM HGSC. The variant annotations are performed using in‐house developed software: HGSC‐SNP‐anno and HGSC‐indel‐anno. Synonymous variants, intronic variants not affecting splicing site, and common benign variants are excluded from interpretation unless they were previously reported as pathogenic variants. The variants were interpreted according to ACMG guidelines (Richards et al. 2015) and patient phenotypes. Variants related to patient phenotypes are usually confirmed by Sanger sequencing for patients and if available, parents. Sanger confirmation is noted in the “References/Comments” section of the tables if performed. It should be noted that the data interpretation are based on our current understanding of genes and variants at the time of reporting.
4. Pharmacogenetic variants are limited to CYP2C9*2, CYP2C9*3, CYP2C9*5, CYP2C9*6,* VKORC1‐1639G>A*, CYP2C19*2, CYP2C19*3, CYP2C19*4, CYP2C19*5, CYP2C19*8, CYP2C19*10, and CYP2C19*17.” Sanger confirmation for pharmacogenomics variants was not routinely done.

For each patient with a reported PGx finding, a pharmacist reviewed the PGx results and patient's current medication usage documented in the electronic medical record (EMR) to provide a clinical interpretation in a pharmacy eConsult. Multiple resources were consulted for reviewing each genotype and gene‐drug relationship including the Clinical Pharmacogenetics Implementation Consortium (CPIC) guidelines (https://cpicpgx.org), UpToDate (https://www.uptodate.com), Micromedex (http://www.micromedexsolutions.com) and AskMayoExpert (Cook et al. 2015). Thus, for drug‐gene relationships that lacked CPIC guidelines, multiple resources were consulted and reviewed to assess their relevance prior to providing recommendations. These recommendations were documented in the patient's EMR to serve as a resource for the medical geneticist to act upon.

### *CYP2C9* variant allele frequencies

The variant allele frequencies were calculated from the Exome Aggregation Consortium data (Lek et al. 2016) for each population represented in the data as well as from a Qatari population using recently published data (Fakhro et al. 2016). The *1 allele (wildtype) was calculated by subtracting the sum of the variant alleles from 1.

## Results

**Figure 1:**
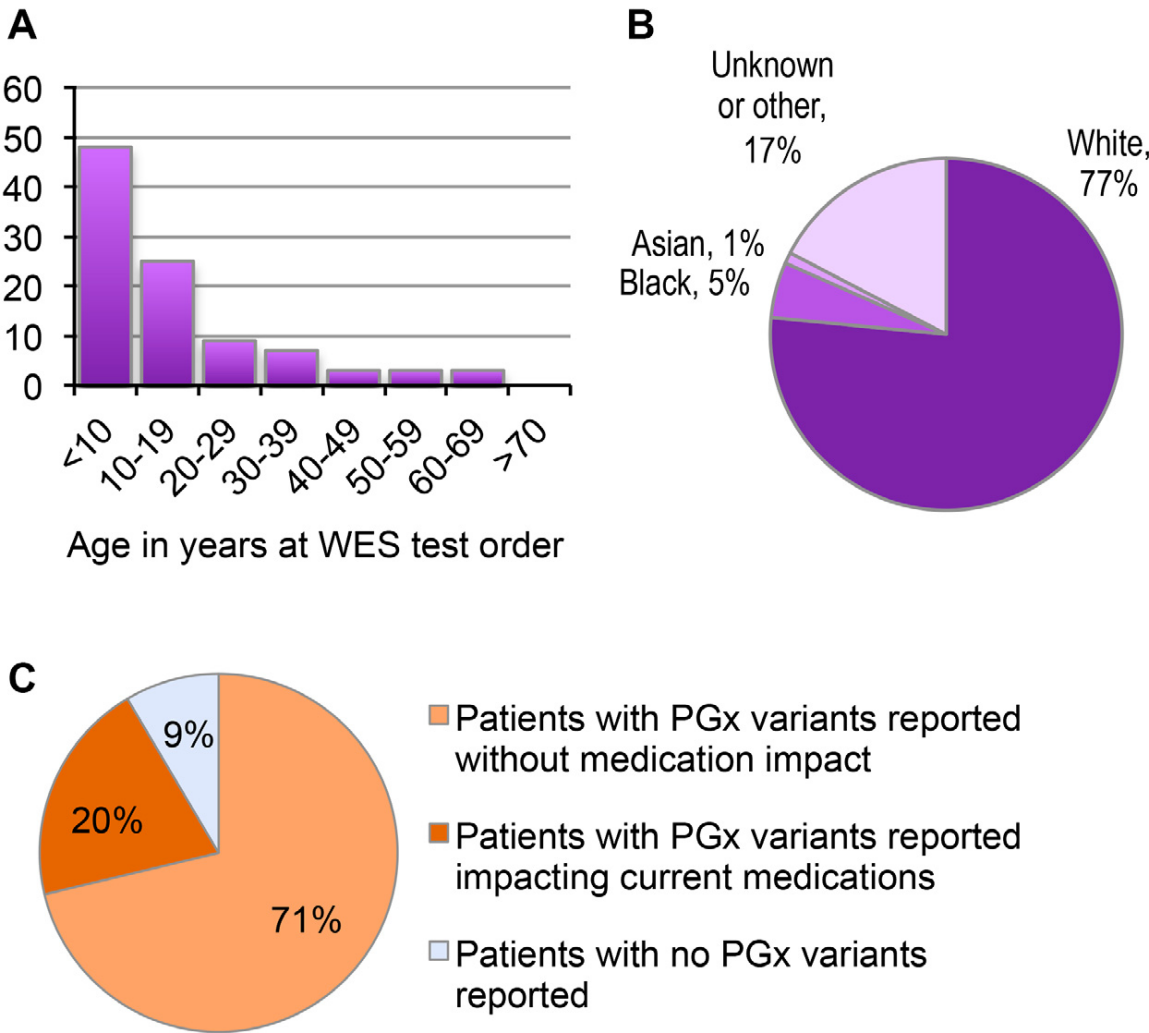
Diagnostic odyssey cohort characteristics. The cohort of 98 patients is skewed toward a pediatric population and is predominantly white by self‐report. (A) Frequency distribution of patients by age at WES testing. (B) Percentage of patients by race as self‐reported and recorded in the EMR. (C) Diagnostic odyssey patients with pharmacogenomic variants reported. A total of 94 patients were evaluated for specific alleles in the *CYP2C19*,*CYP2C9*, and *VKORC1* genes with the majority having at least one variant allele identified.

From September 2012 to November 2015, the Individualized Medicine Clinic saw 98 patients who received clinical WES results for the purpose of identifying the genetic cause of their disease and who could optionally receive secondary PGx results (Lazaridis et al. 2016). This cohort was primarily pediatric (Fig. 1A); the median age at the time of testing was 10 years. A majority of patients had neurological involvement in the disease presentation (71%). Eighty‐eight patients (90%) were taking a total of 609 medications including 237 unique medications. The cohort was 77% white, 5% black, and 1% Asian, with 17% having designated their race as “unknown” or “other”, and one patient having not disclosed race (Fig. 1B). Importantly, during review of the patients’ pedigrees and family histories it was determined that those patients who self‐identified as “unknown” or “other” were of Middle Eastern ancestry.

The reported PGx variant alleles included only *CYP2C19* (MIM:124020) alleles: *2, *3, *4, *5, *8, *10, and *17,* CYP2C9* alleles: *2, *3, *5, and *6, and the *VKORC1* (MIM:608547) allele: c.‐1639G>A. For the *CYP2C19* and *CYP2C9* genes, *1 was not a reported allele by the Baylor Genetics WES test. Three patients declined PGx variant reporting, and for one patient, the clinical report stated PGx results could not be returned due to technical reasons. A total of 94 patients were evaluated for the PGx variant alleles listed above, of which, 91% had one or more variant alleles identified (Fig. 1C).

A clinical pharmacist reviewed the EMR of each patient with PGx variant alleles reported for current medication usage and made medication management recommendations based on potential gene‐drug interactions. Recommendations were recorded as a clinical note by Pharmacy, and are accessible to any prescribing physician. Nineteen patients (20%) received recommendations for their current medication use as a result of the PGx variant alleles reported in their clinical WES test (Fig. 1C).

**Figure 2:**
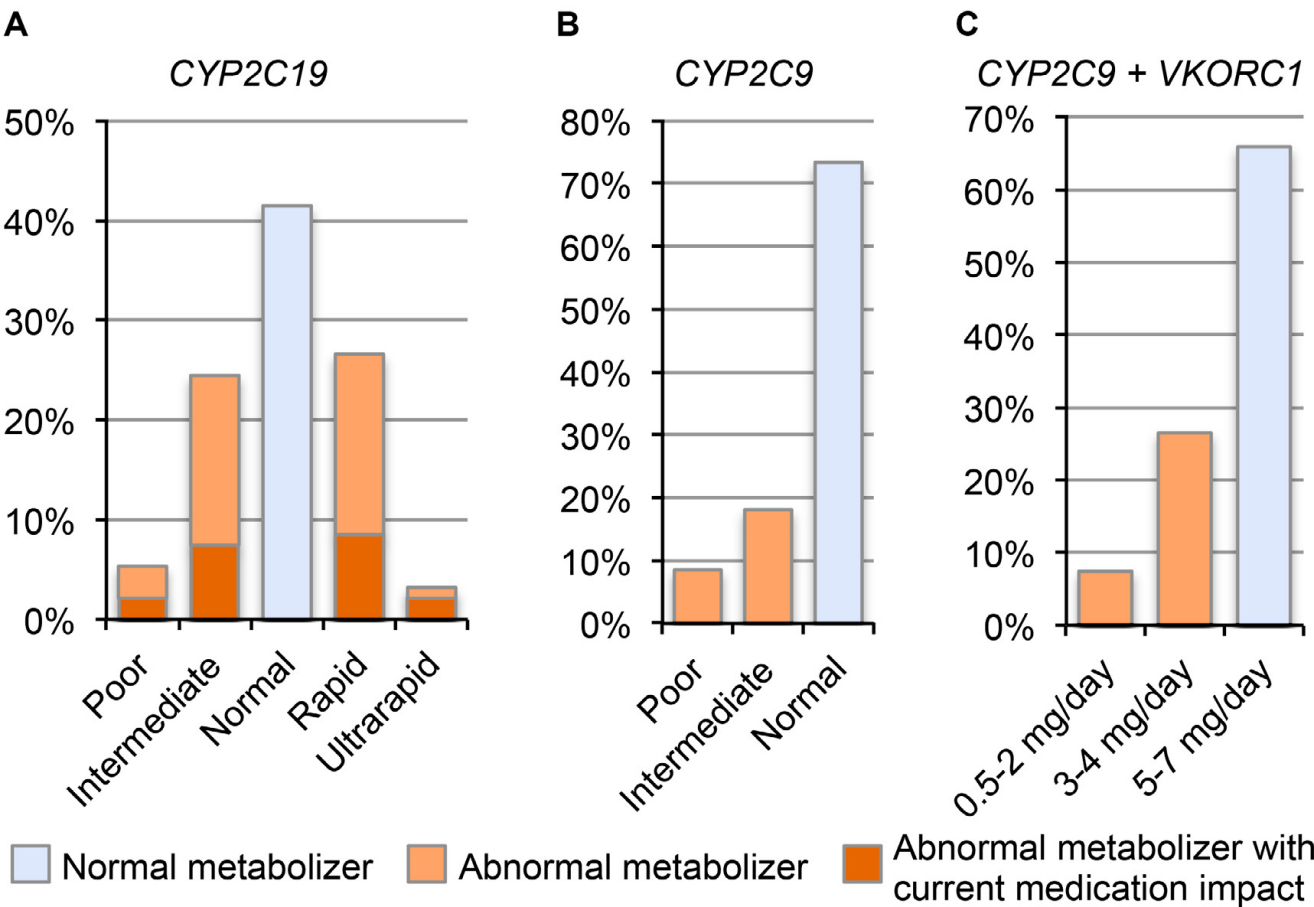
Patients by metabolizer phenotype or warfarin dosing recommendations. A total of 94 patients were evaluated for PGx variant alleles by Baylor Genetics as part of clinical WES testing. The metabolizer phenotypes were interpreted from the variant alleles reported for (A) *CYP2C19* (NG_008384.2; NC_000010.10; NM_000769.2; Build GRCh37.p13) and (B) *CYP2C9* (NG_008385.1; NC_000010.10; NM_000771.3; Build GRCh37.p13), and the suggested warfarin dosing recommendations for the (C) combined interpretation of *CYP2C9* and *VKORC1* (NG_011564.1; NC_000016.9; NM_024006; Build GRCh37.p13) according to CPIC guidelines. The *1 allele was inferred in the absence of a reported variant allele. Current medication use was abstracted from the EMR.

Cytochrome P450 2C19 (CYP2C19) metabolizes medications including proton‐pump inhibitors (PPIs), antiepileptics, and the antiplatelet medication clopidogrel, among others. For *CYP2C19*, of the seven variant alleles reported by the testing facility, only the *2 and *17 alleles were identified in our cohort. The *2 variant is a loss‐of‐function allele and *17 an increased activity allele. The Baylor Genetics clinical test reports only specific variant alleles when identified in a patient and, therefore, the *1 allele (wildtype) was inferred in the absence of a reported variant allele. For example, a single heterozygous *2 variant identified in *CYP2C19* was interpreted as the patient being the *1/*2 genotype. Likewise, a patient with no *CYP2C19* variants reported was interpreted as being the *1/*1 genotype. The corresponding drug metabolism phenotype for each genotype, according to the 2016 CPIC term standardization (Caudle et al. 2016), is shown in Table 1. A distribution of each drug metabolism phenotype across the patient cohort is illustrated in Fig. 2A. Of the 94 patients evaluated for these PGx alleles, 41% were classified as normal, 26% as rapid, 3% as ultrarapid, 24% as intermediate, and 5% as poor metabolizers for *CYP2C19*. These percentages are consistent with those in the 2013 CPIC guidelines (Scott et al. 2013). The medications for which management recommendations were made by the pharmacist, mainly consisting of antiepileptics, anticonvulsants, and PPIs, are shown in Table 1.

**Table 1:** *CYP2C19* and *CYP2C9* genotypes, phenotypes, and actionable medications

Metabolizer phenotype	*CYP2C19* genotype	Total	% of cohort	Patients with medication impact	Medications
Ultrarapid metabolizer	*17/*17	3	3%	2	Omeprazole, esomeprazole
Rapid metabolizer	*1/*17	24	26%	8	Clobazam, diazepam, lacosamide, omeprazole, sertraline,
Normal metabolizer	*1/*1	39	41%	NA	NA
Intermediate metabolizer	*1/*2	19	20%	6	Citalopram, clobazam, diazepam, esomeprazole, omeprazole, sertraline
	*2/*17	4	4%	1	Omeprazole
Poor metabolizer	*2/*2	5	5%	2	Diazepam, sertraline

Metabolizer phenotype	*CYP2C9* genotype	Total	% of cohort	Patients with medication impact	Medications
Normal metabolizer	*1/*1	69	73%	NA	NA
Intermediate metabolizer	*1/*2	13	14%	1	No patients were taking medications with actionable or informative PGx recommendations for *CYP2C9* variants
	*1/*3	4	4%	0	
Poor metabolizer	*2/*2	2	2%	0	
	*2/*3	5	5%	0	
	*2/*6	1	1%	0	

*CYP2C19*: NG_008384.2; NC_000010.10; NM_000769.2; Build GRCh37.p13. *CYP2C9*: NG_008385.1; NC_000010.10; NM_000771.3; Build GRCh37.p13. NA = not applicable.

Cytochrome P450 2C9 (CYP2C9) metabolizes nonsteroidal anti‐inflammatory drugs (NSAIDs), antiepileptics, and the anticoagulant warfarin, among other medications. Of the four variant alleles reported by the testing facility, only *2, *3, and *6 variant alleles were identified in our cohort. Similarly to the genotype interpretation for *CYP2C19*, we inferred the *1 allele is present for *CYP2C9* in the absence of a reported variant allele. The inferred genotypes and interpreted metabolism phenotypes are shown in Table 1. Of the 94 patients evaluated for PGx variant alleles, 73% were normal, 18% were intermediate, and 8% were poor metabolizers. No patients were currently taking medications impacted by the *CYP2C9* variant alleles reported (Fig. 2B and Table 1).

Vitamin K epoxide reductase complex subunit 1 (*VKORC1*) is responsible for reducing and activating vitamin K and thereby allowing blood clot formation. The anticoagulant, warfarin, is an antagonist of this enzyme and its efficacy of anticoagulation is decreased by a polymorphism in the promoter of *VKORC1* (c.‐1639G>A). Heterozygous and homozygous carriers of this polymorphism were identified in our cohort of patients. Warfarin is primarily metabolized through CYP2C9; therefore, both *VKORC1* and *CYP2C9* PGx variant alleles contribute to warfarin dosing recommendations according to current CPIC guidelines (Johnson et al. 2011). The *VKORC1* and inferred *CYP2C9* genotypes are shown in Table 2 grouped by the warfarin dosing recommendations. The *6 variant allele is interpreted in the same manner as the *3 variant allele, since it is a null allele. These data are summarized in Fig. 2C. The suggested warfarin dosing according to the CPIC guidelines is 5–7 mg/day for 66%, 3–4 mg/day for 27%, and 0.5–2 mg/day for 7% of the 94 patients evaluated for PGx variants.

**Table 2:** *CYP2C9* and *VKORC1* genotypes by warfarin dosing recommendations

Warfarin dose	*VKORC1*;* CYP2C9* genotype	Total	% of cohort
5–7 mg/day	GG; *1/*1	20	21%
	GG; *1/*2	7	7%
	GA; *1/*1	35	37%
3–4 mg/day	GG; *1/*3	2	2%
	GG; *2/*2	1	1%
	GG; *2/*3	1	1%
	GA; *1/*2	4	4%
	GA; *2/*2	1	1%
	AA; *1/*1	14	15%
	AA; *1/*2	2	2%
0.5–2 mg/day	GA; *2/*3	2	2%
	GA; *2/*6	1	1%
	AA; *1/*3	2	2%

No patients with decreased warfarin‐dosing recommendations were taking warfarin at the time the WES results were returned. Genotypes not shown, such as *3/*3 were not observed in our cohort. *CYP2C9*: NG_008385.1; NC_000010.10; NM_000771.3; Build GRCh37.p13. *VKORC1*: NG_011564.1; NC_000016.9; NM_024006; Build GRCh37.p13).

As we interpreted the PGx findings in our cohort and made medication recommendations, we hypothesized that inference of the wildtype (*1) allele for *CYP2C19* and *CYP2C9* may not always be accurate. There are other actionable variant alleles identified in these genes that are not included in the subset of variant alleles reported in the clinical test ordered for these patients. Consequently, it is possible we may incorrectly infer a *1 allele when, in fact, a patient has one of these nonreported actionable variants. The inference of the *1 allele and the metabolizer phenotype interpreted from this genotype could then lead to inappropriate medication recommendations.

**Figure 3:**
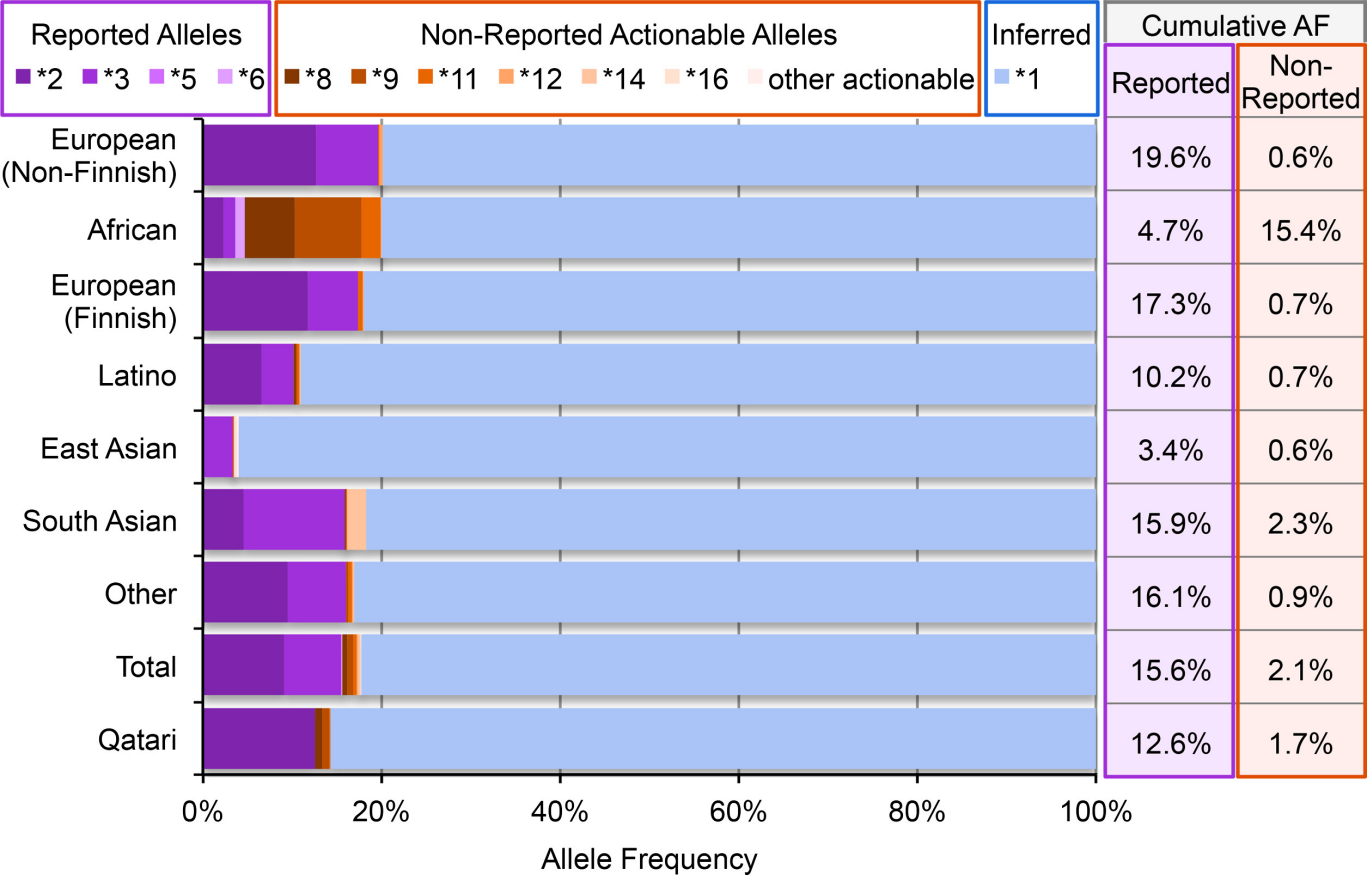
Allele frequencies for variant alleles in *CYP2C9* and inferring *1. Variant allele frequencies for *CYP2C9* (NG_008385.1; NC_000010.10; NM_000771.3; Build GRCh37.p13) differ by population as calculated from publicly available data (Fakhro et al. 2016; Lek et al. 2016) leading to varying probability of error in inferring the presence of a *1 allele when only a subset of alleles are reported. Purple shading indicates variant allele frequencies from the variant alleles reported clinically on the cohort. Orange shading indicates variant allele frequencies from actionable alleles not reported on the cohort. Blue shading indicates the frequency of alleles inferred as being *1 in the absence of the variant alleles.

To assess the likelihood of an incorrect *1 inference, we determined the allele frequencies for the reported alleles for *CYP2C9* by the testing facility (*2, *3, *5, and *6) as well as the nonreported, but actionable alleles including*4 (Sullivan‐Klose et al. 1996), *8, *9 (Blaisdell et al. 2004), *11–17 (DeLozier et al. 2005), *25, *26, *28, *30, and *33 (http://www.cypalleles.ki.se/cyp2c9.htm) using population specific data from the Exome Aggregation Consortium (ExAC) (Lek et al. 2016) (Fig. 3 and Table S1). We chose to evaluate *CYP2C9* because all actionable alleles are in the coding regions and covered in the ExAC sequencing data. The allele frequencies vary between populations with *2 having the largest non‐wildtype frequency overall. In the European (non‐Finnish) population, the total frequency of actionable alleles not reported by the testing facility is 0.58%. In the African population, however, the total frequency of actionable alleles not reported by the testing facility is 15.4%. Reporting only the alleles from the clinical test used here, 16% of what would be the inferred *1 alleles would actually be one of the actionable alleles not reported by the WES test. Consequently, for patients of African descent, inferring a *1 allele in the absence of a reported variant allele from the clinical WES test has a substantial probability of being incorrect.

We also determined the variant allele frequencies from a Qatari population using recently published data (Fakhro et al. 2016), since nearly 17% of our cohort is of Middle Eastern descent. Only four of the actionable alleles we assessed were identified in *CYP2C9* in the Qatari population data, and include *2, *8, *9, and *11 (Fig. 3 and Table S1). For this population, the nonreported actionable alleles accounted for 1.7% of alleles, which could be incorrectly inferred as *1 in the clinical WES test.

## Discussion

Pharmacogenomics (PGx), the study of how genetic variation may inform medication response, has been reported in a cohort of diagnostic odyssey patients and may be informative to the clinical care of this population. The reported genes (*CYP2C19*,* CYP2C9*, and *VKORC1*) and genotypes predict a patient's response to the well‐known medications warfarin and clopidogrel, but in addition, *CYP2C19* and *CYP2C9* are critical to the activation and clearance of at least 23 other medications (https://cpicpgx.org/genes-drugs/). Thus, the appropriate interpretation of these findings is key to individualizing medication prescription, avoiding medication toxicity, and maximizing therapeutic response.

The interpretation, communication, and management of these results, however, are not without unique challenges. Because PGx results are only informative when particular medications are used, it is imperative they are interpreted in the context of the patient's medication needs. To be clinically actionable, they must be readily available at the time of medication prescription or review. As such, PGx results may be informative not only at the time the clinical test report is returned, but also at any future time the patient is prescribed new medications. Furthermore, additional gene‐drug interactions are likely to be discovered, requiring PGx results to be actively maintained and dynamically interpretable.

There are both technical and clinical barriers to the appropriate access and use of PGx results. Considerable effort has been made to implement clinical decision support (CDS) systems with automatic alerts to notify a prescribing physician when a relevant gene‐drug interaction is present for a patient and educational components to assist the clinician with understanding the alerts (Arwood et al. 2016; Caraballo et al. 2016; Hicks et al. 2016; Hoffman et al. 2016; Manzi et al. 2016; St Sauver et al. 2016). Even with these systems, however, barriers to successful and efficient integration of PGx results exist. The clinical reports for our patients are pdf files generated from an outside institution scanned into the patient's EMR, which does not allow the PGx CDS system in the institution to create alerts from the findings. To ensure the prescribing physicians have access to the PGx results for their patients, a pharmacy consult was conducted for each patient with PGx findings. Without added steps to highlight these secondary findings, institutions could be at significant risk and liability for mismanagement of their patients by failure to recognize PGx results in the medical record.

Importantly, 20% of the 94 patients evaluated for PGx variants were taking a medication potentially impacted by the PGx finding. The majority of the patients in our cohort were pediatric with neurological involvement, often including seizures, behavioral disorders, developmental delay, or intellectual disability. The majority of the prescribed medications with relevant PGx results were for the management of gastroesophageal reflux or seizure disorders; eight patients were taking diazepam and seven patients were taking omeprazole. The other medications with potential PGx variant impact included citalopram, clobazam, esomeprazole, lacosamide, and sertraline. Of the 98 patients who received clinical exome sequencing for a suspected genetic disorder, 21 patients (21%) had seizures included in their primary reason for referral.

There are, however, limitations to the interpretation of the PGx results presented in this cohort. The drug metabolism pathways are complex and often more than one PGx gene is involved in the metabolism of a particular medication. When only a subset of PGx genes are tested, the pharmacist is limited in what medication management recommendations can be made. For example, 15 individuals were taking diazepam, of which, eight had variant alleles identified in *CYP2C19* that may influence the efficacy or toxicity. However, diazepam is a major substrate of both *CYP2C19* and *CYP3A4* (MIM:124010) that metabolize it into the active metabolites, N‐desmethyldiazepam, temazepam, and oxazepam, and depending on the rate of the production of these metabolites, efficacy, and toxicity can be affected (Whirl‐Carrillo et al. 2012). Therefore, a full understanding of the genetic influence on the efficacy and toxicity of diazepam can only achieved by evaluating the genetic variation in both genes.

While 91% of patients in this study had at least one PGx variant reported in their clinical WES results, expanding the number of genes being tested by only three, would increase the number of patients with reported variants to nearly 100%, according to a recent study (Ji et al. 2016). And, of the 88 patients (90%) taking medications in the cohort, 60 patients were prescribed a medication with known potential gene‐drug interactions, suggesting the utility of expanded testing in this population of patients. If we were to pursue PGx testing based on individual medication usage and according to the current actionable gene‐drug pairs for drug metabolizing genes used at Mayo Clinic (Cook et al. 2015), 43 patients would be tested for *CYP3A4/5*, 35 for *CYP2C19*, 22 for *CYP2D6*, 8 for *CYP1A2* (MIM:124060), and 9 for *CYP2C9*. A recent study from the NIH Undiagnosed Diseases Program (Lee et al. 2016) also established that PGx results were informative for guiding therapy in their cohort of 308 families. Lee and colleagues evaluated single nucleotide changes that have been reported to impact drug efficacy based on Pharmacogenomics Knowledgebase (PharmGKB). They report 9 patients with potential gene‐drug interactions including the genes: *HTR2C* (MIM:312861), *EPHX1* (MIM:132810), *OPRM1* (MIM:600018), *F13A1* (MIM:134570), and *NOS3* (MIM:163729). As the cost of testing continues to decrease, it may be reasonable to expand the breadth of genetic testing for these patients to include more or “all” of the PGx genes.

A common challenge of PGx test interpretation is inferring the presence of the wildtype, or *1, allele, in the absence of a reported result. As we show with the allele frequencies of *CYP2C9* across different populations, not reporting all actionable variants could lead to incorrectly inferring a *1 allele when an individual actually carries a nonreported but actionable allele. In the African population in the ExAC data, 15.4% of alleles are actionable but not reported when only reporting *2, *3, *5, and *6 variant alleles. That means on average 16.0% of inferred *1 alleles for this population are incorrect. Recommending additional variant testing may be warranted for individuals from this population if *CYP2C9* metabolizer status is important to medications the patient may need. This limitation has been described with regard to warfarin dosing recommendations for the African American population and making dosing predictions without including the common African genotypes was associated with inappropriate dosing (Cavallari et al. 2010; Drozda et al. 2015).

The inference of *1 alleles is also potentially problematic for patients from populations who are underrepresented in terms of genetic sequence data. Approximately 17% of our patients are of Middle Eastern descent. There are limited large sequence datasets that include individuals of Middle Eastern descent; consequently, reference databases like ExAC have limited information on these populations. This lack of data makes interpretation of genetic results from individuals with these ethnicities challenging. Analysis of recent data from a Qatari population (Fakhro et al. 2016) as well as from East Asian and Latino populations in the ExAC database (Lek et al. 2016) identified fewer of the known PGx alleles. Further study of the variation present in specific populations contributing to drug metabolism phenotypes will improve our ability to interpret PGx results for these individuals.

Pediatric medication dosing is often difficult to determine due to the paucity of clinical studies focusing on children and the difficulty of translating recommended adult dosing paradigms into pediatric care (Leeder et al. 2014). Although total body size is a contributing factor to achieving appropriate active medication levels, other factors may impact drug response, including body composition, body proportions, and age‐related differences in gene expression profiles throughout human development. Pharmacodynamics, pharmacokinetics, and subsequent pharmacogenomic studies are challenging to conduct in children (van den Anker et al. 2011; Neville et al. 2011; Kearns and Artman 2015). Pharmacokinetics is heavily driven by drug metabolism and our understanding of the development of the drug‐metabolizing enzyme system from birth to adulthood is incomplete (Koukouritaki et al. 2004). *CYP2C* enzyme expression is activated around the time of birth with enzyme levels at ~30% of adult levels in the first year of life and is largely comprised of *CYP2C9* (Hines and McCarver 2002). The transition of the *CYP2C* expression to adult levels throughout childhood is poorly understood (Treluyer et al. 2000; Hines and McCarver 2002). This is further complicated by recent studies showing that CYP protein expression and enzyme activity can be discordant (Sadler et al. 2016). While we understand that these differences by age and stage of development exist, contributing to therapeutic variability, our ability to predict the appropriate dosing requirements by these developmental differences is understudied and limited (Hines and McCarver 2002).

The nuances to interpreting an individual's PGx results and determining their relevance in the context of the many intrinsic and extrinsic factors also contributing to the efficacy or toxicity of a medication to meet the individual's therapeutic needs is indeed a complex undertaking. While the technologies enabling the identification of genetic variation get better and PGx testing becomes more affordable and more widely adopted, our understanding of the meaning of this genetic variation will continue to improve. With it, the CDS systems that notify physicians of gene‐drug interactions when making the prescription will continue to expand and be refined. A pharmacist trained in PGx may remain a key individual, however, for integrating PGx into the complexities of pharmacotherapy. Intrinsic factors such as age, body size, disease state, lifestyle choices, and medication compliance must be addressed alongside any potential gene‐drug or drug‐drug interactions and consideration of possible medication delivery routes. The pharmacist can make recommendations to maximize the therapeutic goals of the physician by addressing the limitations and complexities of the individual patient, including their PGx genotype. This type of consult may be particularly beneficial for patients on a diagnostic odyssey of which the majority are children taking many medications for complex symptomatology often as part of a poorly defined disease. Additionally, while we describe the reactive interpretation and impact on current medication use in this population, these results will continue to inform therapeutic strategies proactively for the patient's lifetime. For maximal efficacy of PGx testing to be realized, then, early proactive and comprehensive testing with EMR CDS integration of results is ideal.

In this study, we describe the secondary PGx findings from clinical WES testing in a cohort of patients seeking a genetic diagnosis for a suspected Mendelian disease. We show that a significant proportion of this mostly pediatric population had actionable PGx results based on their current medication use. The likely benefit of these results on patient medication management suggests continued, and potentially expanded, PGx testing in this population is warranted. However, it is important to be cognizant of the limitations inherent in PGx testing, as well as the complexities of result interpretation and data management. It is imperative that health‐care institutions are aware of such secondary findings and take steps to ensure that PGx findings are properly integrated into the patient's medical record. Such steps should ensure all future prescriptions are properly informed by the PGx findings and recommendations dynamically reflect the continued expansion of PGx knowledge. Because of these challenges, we highlight the need for conscientious interpretation and management of the PGx results to ensure appropriate prescribing decisions can be made with regard to current as well as any future medication needs.

## Conflict of Interest

No authors have any relevant disclosures or potential conflicts of interest related to the content of this manuscript. MAC and ETM contributed to the design, data collection, data interpretation, and to preparation and review of the manuscript. NJB, PRB, TMM, TMK, DB, KNL, and EWK contributed to the design, data interpretation, and to the preparation and review of the manuscript.

## Supporting information


**Table S1** Allele frequencies of the actionable variant alleles for CYP2C9 calculated from the publicly available data.
