# Supplementary material for: Pharmacogenomic findings from clinical whole exome sequencing of diagnostic odyssey patients
Source: Mol Genet Genomic Med. 2017 Mar 19;5(3):269–79. doi: 10.1002/mgg3.283 (PMC5441410; doi:10.1002/mgg3.283)
Supplement: Supplementary file 1 — Table S1 Allele frequencies of the actionable variant alleles for CYP2C9 calculated from the publicly available data. [file MGG3-5-269-s001.docx]

|  | **CYP2C9 allele (NM_000771.3)** | **Nucleotide**  **change** | **Amino acid**  **change** | ***Allele** | **European (Non-Finnish)** | **African** | **European (Finnish)** | **Latino** | **East**  **Asian** | **South**  **Asian** | **Other** | **Total** | | **Qatari†** |
| --- | --- | --- | --- | --- | --- | --- | --- | --- | --- | --- | --- | --- | --- | --- |
| **Reported** | CYP2C9*2 | 430C>T | R144C | *2 | 12.68% | 2.35% | 11.70% | 6.60% | 0.03% | 4.60% | 9.47% | 9.14% | | 12.56% |
|  | CYP2C9*3 | 1075A>C | I359L | *3 | 6.88% | 1.26% | 5.63% | 3.58% | 3.38% | 11.31% | 6.61% | 6.37% | | 0.00% |
|  | CYP2C9*5 | 1080C>T | D360E | *5 | 0.00% | 0.00% | 0.00% | 0.00% | 0.00% | 0.01% | 0.00% | 0.00% | | 0.00% |
|  | CYP2C9*6 | 818delA | 273fs | *6 | 0.00% | 1.05% | 0.00% | 0.04% | 0.00% | 0.00% | 0.00% | 0.09% | | 0.00% |
|  | | | | | | | | | | | | | | |
| **Not reported, but actionable** | CYP2C9*8 | 449G>A | R150H | *8 | 0.03% | 5.60% | 0.00% | 0.22% | 0.01% | 0.06% | 0.00% | | 0.52% | 0.76% |
|  | CYP2C9*9 | 752A>G | H251R | *9 | 0.02% | 7.54% | 0.00% | 0.19% | 0.01% | 0.01% | 0.22% | | 0.67% | 0.81% |
|  | CYP2C9*11 | 1003C>T | R335W | *11 | 0.22% | 2.14% | 0.54% | 0.16% | 0.01% | 0.19% | 0.33% | | 0.38% | 0.15% |
|  | CYP2C9*12 | 1465C>T | P489S | *12 | 0.30% | 0.06% | 0.15% | 0.10% | 0.00% | 0.02% | 0.22% | | 0.19% | 0.00% |
|  | CYP2C9*14 | 374G>A | R125H | *14 | 0.01% | 0.01% | 0.00% | 0.07% | 0.01% | 2.04% | 0.11% | | 0.30% | 0.00% |
|  | CYP2C9*16 | 895A>G | T299A | *16 | 0.00% | 0.00% | 0.00% | 0.00% | 0.35% | 0.00% | 0.00% | | 0.02% | 0.00% |
|  | CYP2C9*4 | 1076T>C | I359T | *4 | 0.00% | 0.01% | 0.00% | 0.00% | 0.00% | 0.00% | 0.00% | | 0.00% | 0.00% |
|  | CYP2C9*13 | 269T>C | L90P | *13 | 0.00% | 0.00% | 0.00% | 0.00% | 0.20% | 0.00% | 0.00% | | 0.01% | 0.00% |
|  | CYP2C9*15 | 485C>A | S162X | *15 | 0.00% | 0.00% | 0.00% | 0.00% | 0.00% | 0.00% | 0.00% | | 0.00% | 0.00% |
|  | CYP2C9*17 | 1144C>T | P382S | *17 | 0.00% | 0.00% | 0.00% | 0.00% | 0.00% | 0.00% | 0.00% | | 0.00% | 0.00% |
|  | CYP2C9*25 | 353-362del | 118fs | *25 | 0.00% | 0.00% | 0.00% | 0.00% | 0.00% | 0.00% | 0.00% | | 0.00% | 0.00% |
|  | CYP2C9*26 | 389C>G | T130R | *26 | 0.00% | 0.00% | 0.00% | 0.00% | 0.00% | 0.00% | 0.00% | | 0.00% | 0.00% |
|  | CYP2C9*28 | 641A>T | Q214L | *28 | 0.00% | 0.00% | 0.00% | 0.00% | 0.00% | 0.00% | 0.00% | | 0.00% | 0.00% |
|  | CYP2C9*30 | 1429G>A | A477T | *30 | 0.00% | 0.00% | 0.00% | 0.00% | 0.05% | 0.00% | 0.00% | | 0.00% | 0.00% |
|  | CYP2C9*33 | 395G>A | R132Q | *33 | 0.01% | 0.00% | 0.01% | 0.00% | 0.00% | 0.02% | 0.00% | | 0.01% | 0.00% |
|  |  |  |  |  |  |  |  |  |  |  |  | |  |  |
| ***1 inferred from reported alleles** | | | | | 80.43% | 95.34% | 82.67% | 89.78% | 96.59% | 84.08% | 83.92% | | 84.39% | 87.44% |
|  |  |  |  |  |  |  |  |  |  |  |  | |  |  |
| ***1 inferred from all actionable alleles** | | | | | 79.85% | 79.98% | 81.96% | 89.04% | 95.95% | 81.73% | 83.04% | | 82.28% | 85.71% |
|  |  |  |  |  |  |  |  |  |  |  |  | |  |  |
| **Alleles incorrectly inferred as *1 from reported alleles** | | | | | 0.58% | 15.36% | 0.71% | 0.74% | 0.64% | 2.35% | 0.88% | | 2.11% | 1.73% |
