# Supplementary material for: Pharmacogenomic findings from clinical whole exome sequencing of diagnostic odyssey patients
Source: Mol Genet Genomic Med. 2017 Mar 19;5(3):269–79. doi: 10.1002/mgg3.283 (PMC5441410; doi:10.1002/mgg3.283)
Supplement: Supplementary file 2 [file MGG3-5-269-s002.docx]

**Supplemental Table S1.** Allele frequencies of the actionable variant alleles for CYP2C9 calculated from the publicly available data.
